# Implementation of a Novel Electronic Patient-Directed Smoking Cessation Platform for Cancer Patients: Interrupted Time Series Analysis

**DOI:** 10.2196/11735

**Published:** 2019-04-09

**Authors:** Meredith Elana Giuliani, Geoffrey Liu, Wei Xu, Mihaela Dirlea, Peter Selby, Janet Papadakos, Nazek Abdelmutti, Dongyang Yang, Lawson Eng, David Paul Goldstein, Jennifer Michelle Jones

**Affiliations:** 1 Princess Margaret Cancer Centre Toronto, ON Canada; 2 Centre for Addiction and Mental Health Toronto, ON Canada; 3 Division of Medical Oncology and Hematology Department of Medicine University of Toronto Toronto, ON Canada

**Keywords:** neoplasms, smoking cessation, implementation science, quality improvement

## Abstract

**Background:**

Continued smoking in cancer patients undergoing treatment results in significantly higher rates of treatment toxicities and persistent effects, increased risk of recurrence and second malignancy, and increased all-cause mortality. Despite this, routine tobacco use screening and the provision of smoking cessation treatment has yet to be implemented widely in the cancer setting.

**Objective:**

The objective of this study was to implement and evaluate the adoption and impact of an innovative Smoking Cessation e-referral System (CEASE) to promote referrals to smoking cessation programs in cancer patients.

**Methods:**

A patient-directed electronic smoking cessation platform (CEASE) was developed to promote smoking screening and referral and implemented at 1 of Canada’s largest cancer centers. The implementation and evaluation were guided by the Ottawa Model of Research Use. An interrupted time series design was used to examine the impact of CEASE on screening rates, referrals offered, and referrals accepted compared with a previous paper-based screening program. A subsample of smokers or recent quitters was also assessed and compared pre- and postimplementation to examine the effect of CEASE on subsequent contact with smoking cessation programs and quit attempts.

**Results:**

A total of 17,842 new patients attended clinics over the 20-month study period. The CEASE platform was successfully implemented across all disease sites. Screening rates increased from 44.28% (2366/5343) using the paper-based approach to 65.72% (3538/5383) using CEASE (*P<*.01), and referrals offered to smokers who indicated interest in quitting increased from 18.6% (58/311) to 98.8% (421/426; *P*<.01). Accepted referrals decreased from 41% (24/58) to 20.4% (86/421), though the overall proportion of referrals generated from total current/recent tobacco users willing to quit increased from 5.8% (24/414) to 20.2% (86/426) due to the increase in referrals offered. At 1-month postscreening, there was no significant difference in the proportion that was currently using tobacco and had not changed use in the past 4 weeks (pre: 28.9% [24/83] and post: 28.8% [83/288]). However, contact with the referral program increased from 0% to 78% in the postCEASE cohort (*P<*.001).

**Conclusions:**

CEASE is an innovative tool to improve smoking screening and can be implemented in both a time- and cost-effective manner which promotes sustainability. CEASE was successfully implemented across all clinics and resulted in improvements in overall screening and referral rates and engagement with referral services.

## Introduction

### Background

It is widely recognized that smoking cessation will decrease the risk of developing certain cancers [1]. In addition, there is a large body of evidence which demonstrates that continued smoking after a cancer diagnosis leads to significantly higher rates of treatment toxicities and persistent effects [1-7], increased the risk of recurrence and second malignancy [1,3,4,8-11], and increased all-cause mortality [1-3,8-10,12-14]. Unfortunately, 20% to 25% of individuals will continue to smoke after a cancer diagnosis and throughout treatment [15,16].

Screening and advice to patients on smoking cessation can be an effective first step to smoking cessation and increases the likelihood that a patient will attempt to quit and be successful [17]. Over the past 5 years, a number of leading cancer organizations have released policy statements which recommend timely and cost-effective assessment of tobacco use and the provision of smoking cessation assistance for all cancer patients [2,17,18]. In 2016, the National Comprehensive Cancer Network released its Clinical Practice Guidelines in Oncology for Smoking Cessation, which highlighted the importance of smoking cessation and recommended that all cancer patients be evaluated and assessed for smoking status, offered counseling on quitting, and provided with tailored cessation services [18]. In Canada, the CAN-ADAPTT (Canadian Action Network for the Advancement, Dissemination and Adoption of Practice-informed Tobacco Treatment) Clinical Practice Guideline for Smoking Cessation [19] recommends that (1) tobacco use status should be updated for all patients by their health care providers (HCPs) on a regular basis and that HCPs should clearly advise to quit and assess the willingness to begin treatment to achieve abstinence (ask, advise, assess), (2) every tobacco user who expresses willingness to begin treatment to quit be offered assistance (assist), and (3) HCPs conduct regular follow-up to assess response and are encouraged to refer patients to relevant resources as part of the provision of treatment (arrange). Cancer Care Ontario (CCO), an agency which oversees the quality of cancer services in the province of Ontario, conducted an environmental scan to determine the need for a standardized approach of smoking cessation programs within the cancer system and subsequently developed a Smoking Cessation Advisory Committee, which established a plan and provided guidance for the implementation of a smoking cessation program in regional cancer centers within Ontario. In the 2011-2015 Cancer Plan, CCO mandated that all new cancer patients be screened for smoking status and that recent or current smokers be advised to quit and assisted with quitting [20].

Despite the myriad of benefits of smoking cessation to cancer patients [3,11,21-24] and the fact that many newly diagnosed cancer patients are motivated to quit smoking and are open to discussions on how to do this [25-27], there remain significant challenges in terms of the implementation of these recommendations and strategies in oncology care settings [20,28]. Consequently, the majority of cancer patients are not screened for smoking status and/or referred to cessation services [2]. This knowledge-to-practice gap in screening and referrals is likely multifactorial [29] and requires innovative and sustainable approaches that consider the realities of the clinical environment and can efficiently screen and refer large volumes of cancer patients.

### Objective and Specific Aims

The objective of this study was to implement and evaluate the adoption and impact of an innovative Smoking *C* essation *e*-referr*a* l *S* yst*e* m (CEASE) to promote referrals to smoking cessation programs in cancer patients. The specific aims of the study were (1) to facilitate the adoption of CEASE in promoting smoking screening and referral to cessation programs and (2) to evaluate the impact of CEASE on screening and referral patterns. We hypothesized that the CEASE system would be successfully implemented and acceptable to patients, would result in increased screening and referral rates, and would subsequently result in increased interactions with smoking cessation programs.

## Methods

Our project followed the Standards for Quality Improvement Reporting Excellence 2.0 guidelines for study design and analysis [30] and was guided by the Ottawa Model of Research Use (OMRU) [31]. This study was reviewed and approved by the University Health Network Research Ethics Board (#15-8974 CE).

### Context

The Princess Margaret Cancer Centre (PM) is 1 of the 14 regional cancer centers in Ontario and the largest single-site cancer hospital in Canada. It comprises 12 cancer site groups, 26 specialty clinics, and approximately 3000 staff who see over 400,000 patient visits each year. In 2017, there were approximately 18,000 new patients registered at PM.

In an effort to align with CCO recommendations, a paper-based screening program was implemented throughout PM between 2014 and 2016 with the goal that every new patient at PM be screened for smoking status and provided with a smoking cessation referral or resource when appropriate. At the time of clinic registration, newly diagnosed cancer patients were identified by the patient flow coordinator within each clinic and provided with a paper screening form to complete and return. The form queried patients on their smoking habits and their interest in smoking cessation. Following this, the screening form was placed in the chart to be reviewed by an HCP during the appointment. If the patient was interested in cessation programs, a referral sheet for the Nicotine Dependence Clinic (Centre for Addiction and Mental Health), Smoker’s Helpline, or the hospital pharmacy was completed by the HCP. If the patient did not want a referral, an information pamphlet and referral numbers for the Smoker’s Helpline and pharmacy were provided. The program’s performance was monitored by collecting the number of eligible patients screened and offered a referral each month. This program resulted in an average of 55% (range across clinics 10%-90%) of all new patients screened for smoking status, with 60% to 70% indicating interest in quitting at the time of screening, but only 20% of patients who indicated interest in quitting received a referral by their HCP to a smoking cessation program.

Our initial research [32-39] and subsequent implementation activities allowed for the identification of enablers for tobacco screening and referral, which included the CCO framework and mandate, PM leadership support, support from the clinical teams, as well as high motivation from the patients in terms of interest in smoking cessation (potential adopters). However, time constraints in already overloaded oncology clinics as well as a lack of familiarity with cessation resources remained significant barriers that resulted in substandard screening and referral rates even when a patient indicated interest in quitting (practice environment; OMRU stage 1). On the basis of these findings, we have adapted our approach and developed an electronic patient-driven model to enable systematic screening and patient self-referral (CEASE). The CEASE model provided a solution to address the time constraints of overloaded oncology clinics as well as a lack of familiarity with smoking cessation resources by oncology HCPs. Furthermore, engagement of patients in their own health care may have considerable potential to achieve beneficial outcomes and can be an important and effective strategy to target knowledge-to-care gaps [40,41].

### Intervention

**Figure 1:**
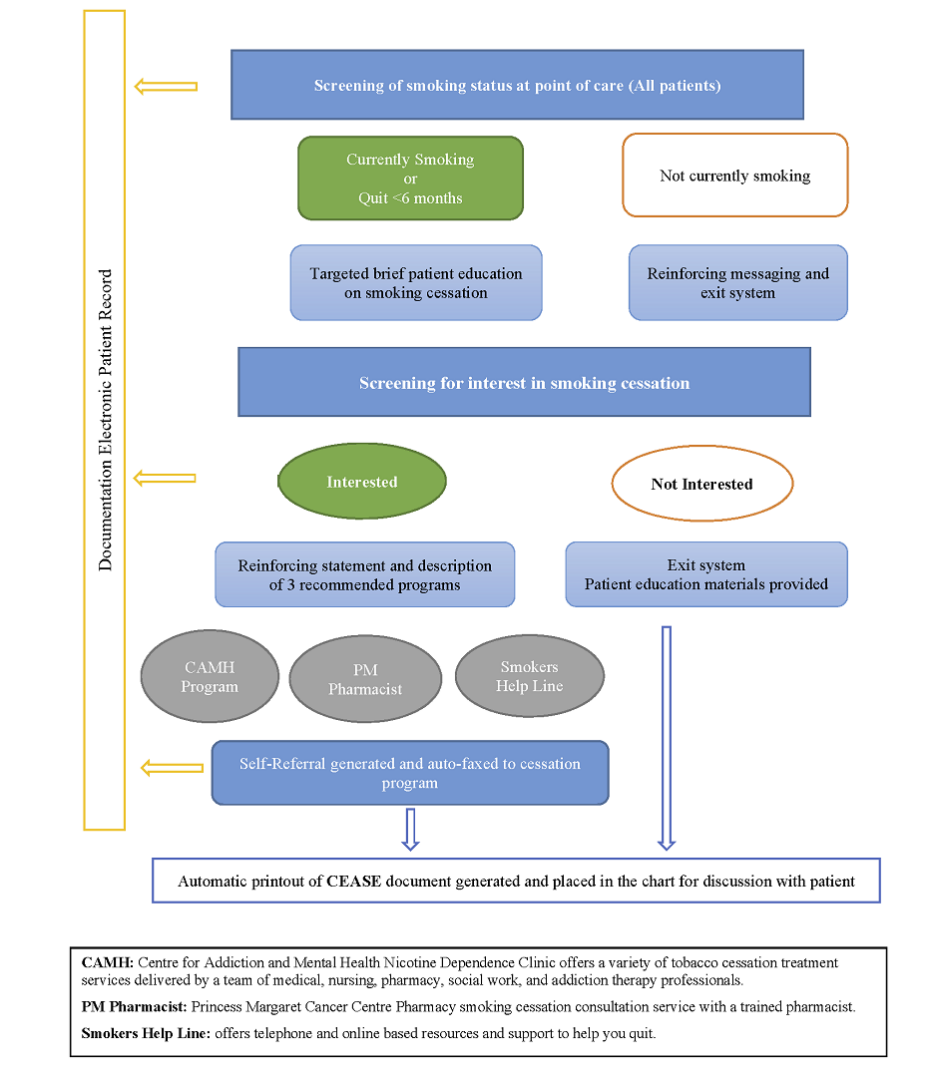
Smoking cessation screening and referral process flow.

CEASE is delivered electronically to newly diagnosed cancer patients on a tablet at the point of care and consists of 3 elements that align with the CAN-ADAPTT Clinical Practice Guideline for Smoking Cessation: (1) a patient-reported smoking assessment tool (ask, assess); (2) brief, standardized patient education regarding smoking (advise), and (3) a simple patient-directed automatic referral system (assist, arrange). On the basis of the screening status (smoker or ecently quit [<6 months] or nonsmoker), a tailored response is generated (see Figure 1). Referrals are automatically sent through the tablet, and the referral sources call within a week to follow-up with the patient. Data from CEASE are archived within the electronic patient record.

### Implementation

To facilitate implementation, we employed multiple enabling and reinforcing strategies based on the Awareness-to-Adherence Model of behavior change [42,43]. The preliminary consultation, diffusion, and dissemination strategy were conducted between July 2015 and October 2015 and included the following strategies to promote *awareness* and *agreement*: (1) gathering feedback from stakeholders (including health care team and patients) on workflow to fine-tune the implementation approach; (2) interviews with patients to gather feedback on the CEASE platform and interface, patient-directed messages, and patient education materials; and (3) presentations to the site teams at weekly tumor boards and rounds (and copy via email) to increase awareness, target attitudes, and to provide an introduction to the CEASE platform and workflow [44]. Following final revisions to the implementation approach, CEASE was rolled out in a step-wise process from October 2015 to January 2016 (initial implementation). Following initial implementation, we employed the following strategies to facilitate *adoption* and *adherence*:

1. Audit and feedback were conducted in each clinic to document if CEASE had been completed in the target population. The performance metrics were then provided to disease site teams (and compared with others) for discussion and to develop solutions to any barriers.
2. Reminders regarding the CEASE program were integrated into routine clinical care team meetings or rounds and sent via email to HCPs as part of the stimulus to the change in practice expected.
3. Information posters were developed through the Cancer Education Program to inform patients about CEASE and encourage them to complete the tablet-based tool. Final workflow and system changes were completed in May 2016.

### Outcomes

#### Process-of-Care Outcomes

An interrupted time series design was used to examine the implementation and impact of CEASE on screening rates, referrals offered, and referrals accepted. The study included 20 monthly intervals: 6 months before implementation (April to September 2015; pre), 8 months during a transition period to accommodate a gradual implementation across all tumor sites (October 2015 to May 2016), and 6 months after full implementation (June 2016 to November 2016; post).

#### Patient-Reported Outcomes

To evaluate the effect of CEASE on subsequent contact with smoking cessation programs and subsequent quit attempts, 1 month following the initial screening (either prepaper screening or postCEASE), a subsample of patients who indicated they were current or recent smokers were sent a follow-up questionnaire to assess uptake of referrals, quit attempts, and reassess smoking status.

### Analysis

Segmented regression was used to assess the impact of the changes on 4 prespecified process-of-care outcomes [44,45]: (1) the proportion screened among all the new patients, (2) the proportion offered referral among total of current smokers and the ones who quit smoking in less than 6 months, (3) the proportion of referral accepted among all the offered referral patients, and (4) the proportion of patients willing to quit among the total of current smokers and the ones who quit smoking in less than 6 months. The segmented regression analysis estimates the interaction terms between the implementation of CEASE and time. As there are 3 time segments, we termed the prepaper screening stage the *pre* (ie, preintervention) period; the second time segment was during the roll-out implementation of CEASE; and the third time segment was after CEASE had been fully implemented across the cancer center, termed *post* for the postintervention period. In the models, the binary regression term *intervention 1* represented the comparison of screening after the start of CEASE (October 2015) versus the *pre*period; the binary regression term *intervention 2* represents the comparison of the *post*period with the time segments before the *post*period.

For each outcome, the segmented regression model had the following form:

Outcome_t_=β_0_+β_1_x Time+β_2_X Intervention 1_t_+β_3_X Time after intervention 1_t_+β_4_X Intervention 2_t_+ Intervention 2_t_+β_5_X Time after intervention 2_t_+ Ɛ_t_

where *Ɛ*_*t*_ is the error term following an auto-regression model adjusting for serial correlation [44,45], and *β*_*0*_ is the intercept for prepaper screening stage, *β*_*0*_*+β*_*2*_ is the intercept for *intervention 1* stage, and *β*_*0*_*+β*_*2*_*+β*_*4*_ is the intercept for *intervention 2* stage, whereas *β*_*1*_ is the slope for prepaper screening stage, *β*_*1*_*+β*_*3*_ is the slope for *intervention 1* stage, and *β*_*1*_*+β*_*3*_*+β*_*5*_ is the slope for *intervention 2* stage. The segmented regression was conducted using PROC AUTOREG in SAS version 9.4 [46].

Prepost, self-reported patient outcome data were compared using Chi-square tests [47] using SPSS version 24.0. In addition, 2-sided tests were conducted, and the statistical significance was set at *P*<.05.

## Results

**Figure 2:**
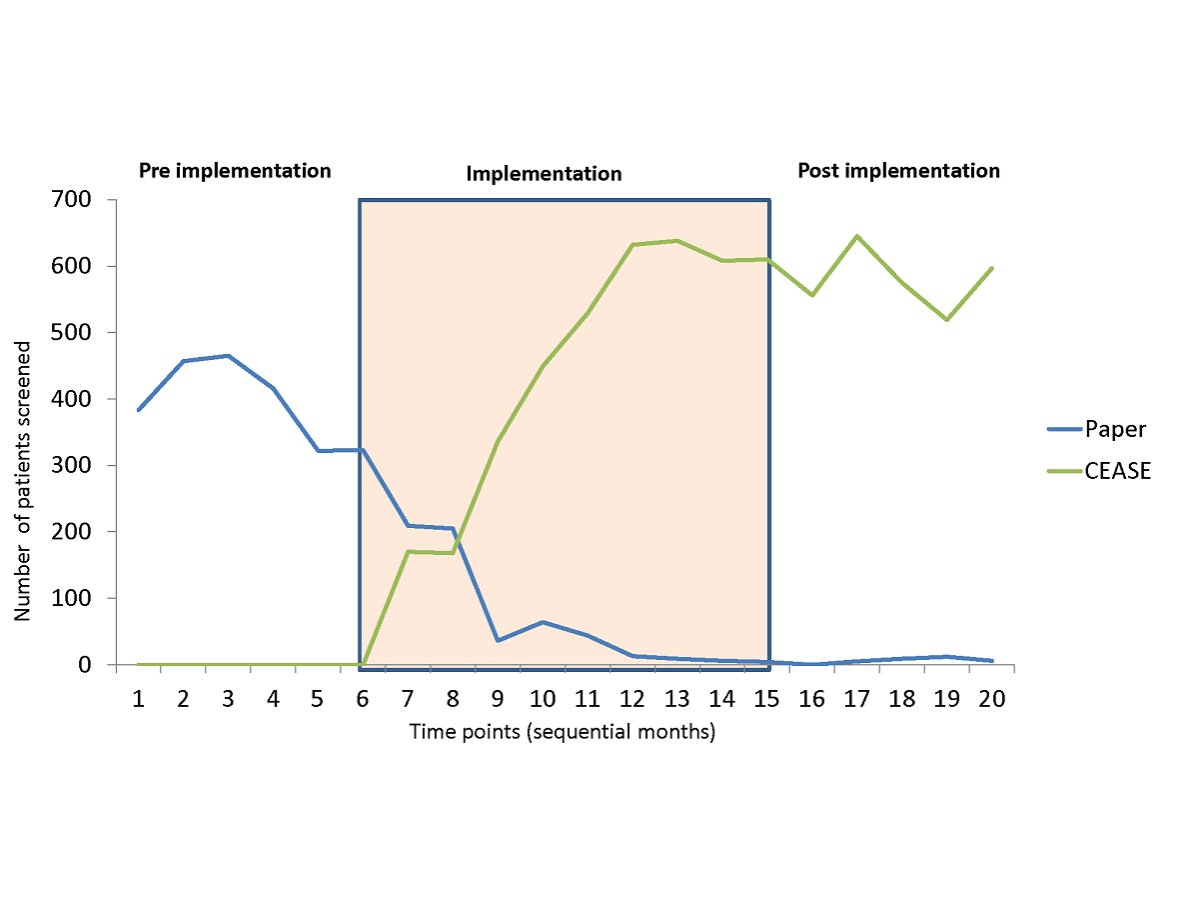
Screening method (paper and CEASE) over time. CEASE: Cessation e-referral System.

A total of 17,842 new patients attended clinics over the study period, including 5343 during the 6-month preintervention period, 7116 during the 8-month implementation period, and 5383 during the 6-month postimplementation period. Figure 2 presents the screening methods used (paper vs CEASE) over the course of the study. By the end of the implementation period and throughout the postimplementation period, only 36 out of the 3538 (1.01%) patients who were screened received the screening on paper.

### Process of Care Outcomes

Multimedia Appendices 1 and 2 present the time series for each outcome indicator, with fitted trends. The final model fit was found to be adequate. The estimated coefficients from the segmented regression analyses are presented in Multimedia Appendix 3. Screening rates increased from preimplementation at 44.28% (2366/5343) using the paper-based approach to 65.72% (3538/5383) at postimplementation using CEASE (*P<*.01). Referrals offered to smokers who indicated interest in quitting increased from 18.6% (58/311) to 98.8% (421/426; *P<*.01). Accepted referrals decreased from 41% (24/58) to 20.4% (86/421), though the overall proportion of referrals generated from total current or recent tobacco users willing to quit increased from 7.7% (24/414) to 20.2% (86/426) due to the increase in referrals offered.

### Pre-Post Self-Report Patient-Reported Data

A total of 29.7% (83/279) of surveys were completed and returned during the preimplementation (paper) phase and 41.9% (288/686) during the postimplementation (CEASE) period. The 2 samples did not differ on any demographic variables. At 1-month postscreening, 24 of 83 (29%) patients in the precohort were still smoking and 83 of 288 (28.8%) patients in the postcohort reported that they were currently using tobacco; 20 of 88 (23%) patients in the precohort and 80 of 288 (27.7%) patients in the postcohort were ok currently using tobacco but had reduced tobacco use over the past 4 weeks; and 37 of 88 (42%) patients in the precohort and 101 of 288 (35.1%) patients in the postcohort reported that they had stopped smoking (pre: 42% and post: 35%). A total of 47% (41/88) of precohort and 76.4% (220/288) of postcohort respondents remembered completing a screening questionnaire about tobacco use at their first visit at PM (*P<*.001). In the precohort, 24% reported receiving a referral from their HCP but none (0%) reported that they had been contacted or had followed up with the referral program. In the postcohort phase, 24% reported that they had accepted a referral through the CEASE program, and of these, 78% had been contacted or followed up with the referral program (*P<*.001).

## Discussion

### Principal Findings

The implementation of CEASE was shown to be feasible and sustainable within a large cancer center with a high volume of patients and supports the use of a technology- and patient-mediated implementation approach. CEASE was also successfully integrated into the electronic medical record in a sustainable fashion. Following implementation, only 1% of patients refused to use the CEASE platform and asked for the paper version, a finding that supports its acceptability. Compared with paper-based screening and HCP-dependent referral, the implementation of the CEASE platform significantly increased screening rates from 44% to 66% and referral rates from 19% to 99%.

It is clear from the recommendations of multiple leading cancer organizations that smoking cessation support is a critical component of a quality cancer program [2,17,18]. Despite this, cancer patients are unlikely to have their smoking systematically assessed and managed [48]. The CEASE platform is able to address all the recommended standards for assessing the smoking status in cancer patients and ensuring that the recommendations for access to cessation services are followed [2]. The platform capitalizes on the use of technology to overcome the most common and problematic barriers to cessation screening [20,28,49] and allows for universal screening and an automatic referral to existing resources and specialized providers. In addition, the use of iPads to deliver the intervention at the point of care, integrated with the hospital electronic medical records, and use of customized logic to personalize responses makes it a highly personalized, efficient, scalable, and sustainable initiative.

We found fairly low interest in participating in formal smoking cessation programs, which has been documented elsewhere and may be due to a number of factors such as low motivation and/or confidence and stigma associated with seeking smoking cessation treatment [46]. Many patients want to quit on their own and, therefore, feel that formal smoking cessation treatment is not needed [50], despite strong evidence that treatment for tobacco dependence is associated with significantly higher long-term quit rates [51]. Interestingly, although screening and referral rates improved significantly with the CEASE program, the proportion of patients who accepted referrals decreased from 41%, when the HCP offered the referral, to 20% when offered through CEASE. Although the reason for this finding is not entirely clear, it is possible that patients are more motivated to quit when they receive this recommendation from their treating oncologist. Advice from an HCP can be a very powerful motivator for behavior change [52]. On the other hand, patients may feel pressured to accept the referral because they worry that it may impact care if they say *no* [53]. Although social desirability is one possible reason for the drop in acceptance of the referral, many patients who refused referrals on the CEASE platform indicated that they would like to quit on their own (they had the option to indicate this). Although the proportion of patients who would like to try quitting on their own likely did not change pre or postimplementation, it may be that HCPs would have discouraged this and tried to provide more support through a referral. Encouragingly, our preliminary postsurvey data found similar overall quit rates between the paper-based and CEASE programs, which suggests that CEASE does not impact quit rates negatively (or positively) despite the higher rate of referral acceptance in the paper-based model. However, the long-term impact of CEASE on cessation rates could not be determined from this study and future work with longer follow-up at 12 months or longer is needed. From a population health perspective, it is important to also note that, despite the finding that less patients accepted a referral through the CEASE system, the overall (absolute) proportion of patients who received a referral actually increased from 8% to 20% due to the fact that all current or recent tobacco users were offered a referral on the CEASE system compared with only 19% of those with the paper-based HCP-dependent system. It is also important to consider that none of the precohort patients who reported receiving a referral through their HCP remembered being contacted or following up with the smoking cessation program, compared with 78% of those who generated their own referral through the CEASE program. A manual system that relies on an HCP to generate and send referrals may seem fairly straightforward, but it requires a clear protocol and workflow, which can be difficult in very busy oncology clinics.

### Strengths and Limitations

The results of this study need to be considered within the context of its limitations. To begin, although we are unaware of any threats to validity, it is possible that events or initiatives outside of the control of the research team occurred at the same time as the intervention, though we are not aware of any other changes to the clinic set-up during the study period or efforts to address smoking screening or cessation. There are also limitations of the platform itself. First, the CEASE intervention was created and implemented in English only. Therefore, the generalizability of these results to patients whose primary language is not English is not known. The electronic interface is amenable to translation, and this would be a valuable future contribution to the literature. In addition, this study was not designed to assess the long-term impact of CEASE on cessation rates, and the follow-up survey was only administered 1 month after screening, which may not allow enough time to properly assess quit rates and attempts. However, it is feasible within the CEASE platform to program reassessments at prespecified time intervals. In this regard, the team recently received approval and implemented routine reassessments of patients who indicated that they were current or recent tobacco users at diagnosis.

Despite these limitations, our study has used an established methodology and knowledge translation framework over a substantial period with a large patient population. To our knowledge, this is the first such study in cancer patients, and the data provided here may guide the development and implementation of smoking cessation screening programs in other cancer and possibly noncancer programs. For example, the CEASE program is now being expanded to the Ontario lung cancer screening program and is being piloted in 2 other cancer programs in Toronto. There has also been interest in noncancer programs (ie, cardiovascular).

### Conclusions

In conclusion, a large majority of newly diagnosed cancer patients are interested in quitting smoking. Electronic, patient-driven screening and referrals via CEASE were successfully implemented across all clinics and resulted in improvements in overall screening and referral rates and engagement with referral services. This represents a sustainable strategy for routine cessation services in cancer care.

## Appendix

Multimedia Appendix 1 Percentage of patients screened and offered a referral.

Multimedia Appendix 2 Percentage of patients who accepted the referral (from those offered a referral) and percentage of referrals generated (from all smokers or current quitters).

Multimedia Appendix 3 Estimated coefficients from segmented regression analyses.

## Abbreviations

CAN-ADAPTT: Canadian Action Network for the Advancement, Dissemination and Adoption of Practice-informed Tobacco Treatment
CCO: Cancer Care Ontario
CEASE: Cessation e-referral System
HCPs: health care providers
OMRU: Ottawa Model of Research Use
PM: Princess Margaret Cancer Centre
