# Supplementary figures and images for: Implementation of a Novel Electronic Patient-Directed Smoking Cessation Platform for Cancer Patients: Interrupted Time Series Analysis
Source: J Med Internet Res. 2019 Apr 9;21(4):e11735. doi: 10.2196/11735 (PMC6477574; doi:10.2196/11735)

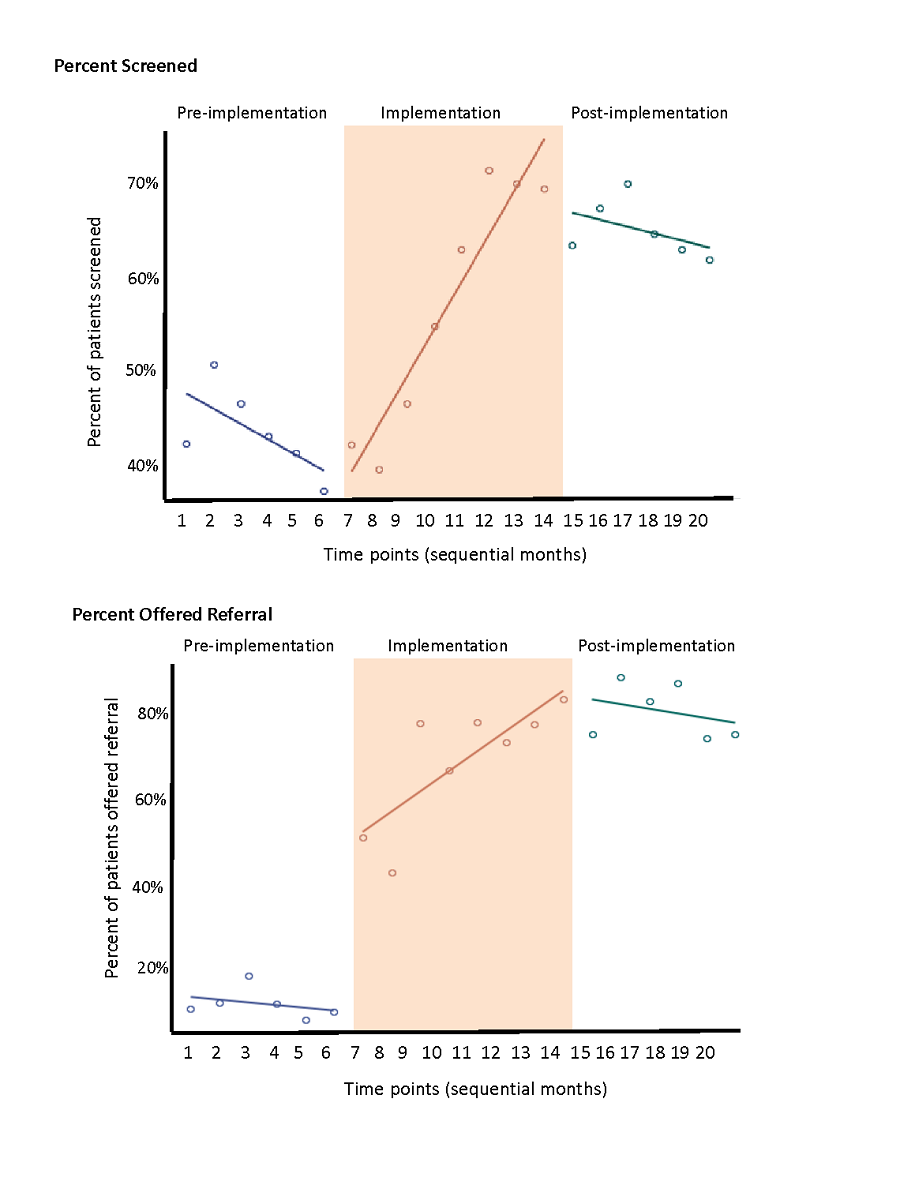

Supplement: Multimedia Appendix 1 [file jmir_v21i4e11735_app1.png]

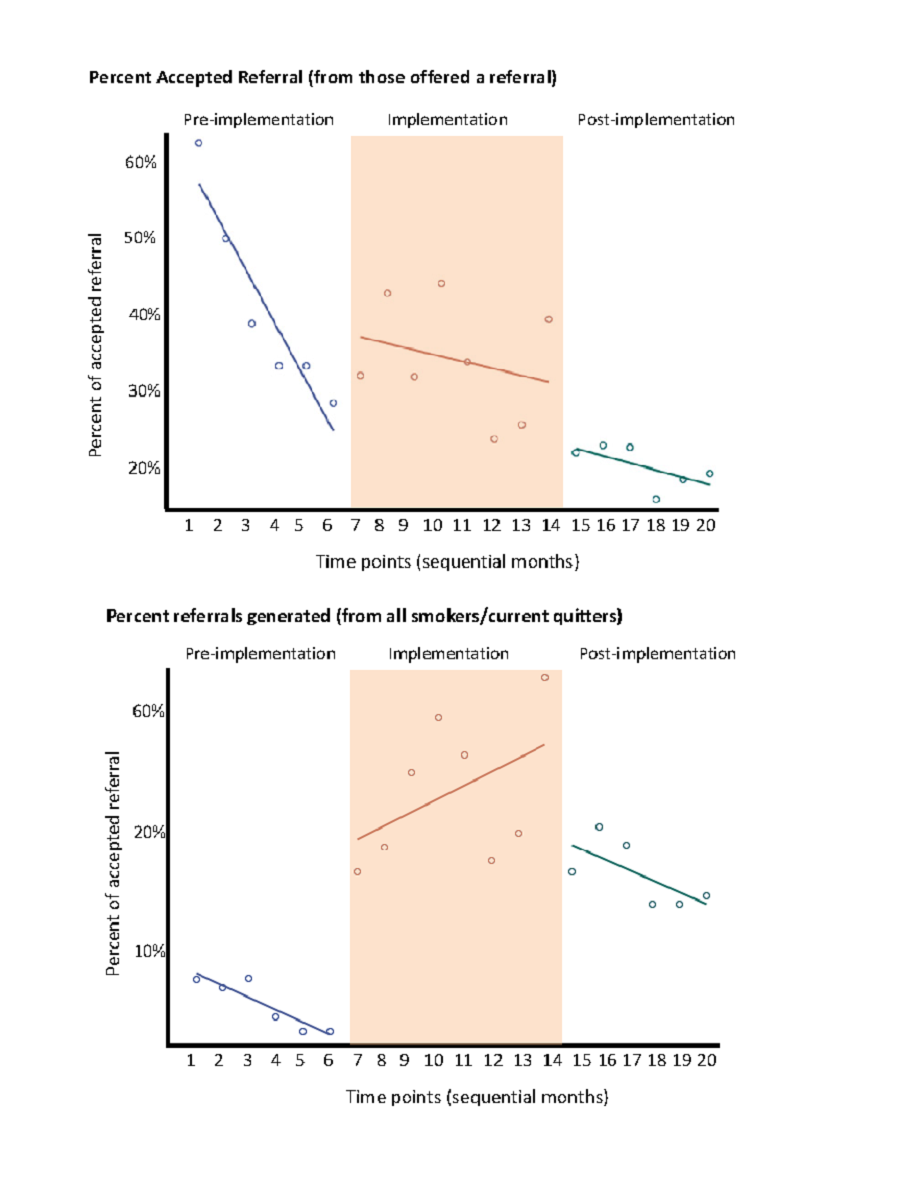

Supplement: Multimedia Appendix 2 [file jmir_v21i4e11735_app2.png]
