# Supplementary material for: Implementation of a Novel Electronic Patient-Directed Smoking Cessation Platform for Cancer Patients: Interrupted Time Series Analysis
Source: J Med Internet Res. 2019 Apr 9;21(4):e11735. doi: 10.2196/11735 (PMC6477574; doi:10.2196/11735)
Supplement: Multimedia Appendix 3 [file jmir_v21i4e11735_app3.docx]

**Results from segmented regression analysis for 4 indicator variables.**

**Outcome A: Total Percent Screened**

**=Total_current_smokers_recent_quitter/total_screened*100**

| Primary Analyses | DF | Estimate | SE | t Value | Pr > \|t\| |
| --- | --- | --- | --- | --- | --- |
| Intercept (baseline rate) | 1 | 16.167 | 1.306 | 12.38 | <.0001 |
| Pre-implementation slope | 1 | 0.403 | 0.335 | 1.20 | 0.2496 |
| Diff_Intercept_12^a^ | 1 | -2.739 | 1.492 | -1.84 | 0.0878 |
| Diff_Slope_12^c^ | 1 | -0.635 | 0.399 | -1.59 | 0.1339 |
| Diff_Intercept_23^b^ | 1 | 1.532 | 1.589 | 0.96 | 0.3514 |
| Diff_Slope_23^d^ | 1 | 0.003 | 0.399 | 0.01 | 0.9948 |

**^a^Diff_Intercept_12 is the difference between the intercepts of interval 1 to interval 2;**

**^b^Diff_Intercept_23 is the difference between the intercepts of interval 2 to interval 3;**

**^c^Diff_Slope_12 is the difference between the slopes of interval 1 to interval 2;**

**^d^Diff_Slope_23 is the difference between the slopes of interval 2 to interval 3.**

The regression for the three intervals are the following:

Interval 1: y = 16.167 + 0.403 time

Interval 2: y = 16.167 + 0.403 time – 2.739 – 0.635 (time – 6) = 17.238 – 0.232 time

Interval 3: y = 16.167 + 0.403 time – 2.739 – 0.635 (time – 6) + 1.532 + 0.003 (time – 14) = 18.728 –0.229 time

None of the slopes and sudden changes are statistically significant.

**Outcome B: Percent Offered Referral**

**= total_offered_referral/total_willing_to_quit*100**

| Primary Analyses | DF | Estimate | SE | t Value | Pr > \|t\| |
| --- | --- | --- | --- | --- | --- |
| Intercept 1 | 1 | 21.7791 | 7.0973 | 3.07 | 0.0083 |
| Slope 1 | 1 | -1.0306 | 1.8224 | -0.57 | 0.5807 |
| Diff_Intercept_12^a^ | 1 | 42.1542 | 8.1075 | 5.20 | 0.0001 |
| Diff_Slope_12^c^ | 1 | 6.9629 | 2.1691 | 3.21 | 0.0063 |
| Diff_Intercept_23^b^ | 1 | -3.8434 | 8.6364 | -0.45 | 0.6631 |
| Diff_Slope_23^d^ | 1 | -6.6659 | 2.1691 | -3.07 | 0.0083 |

**^a^Diff_Intercept_12 is the difference between the intercepts of interval 1 to interval 2;**

**^b^Diff_Intercept_23 is the difference between the intercepts of interval 2 to interval 3;**

**^c^Diff_Slope_12 is the difference between the slopes of interval 1 to interval 2;**

**^d^Diff_Slope_23 is the difference between the slopes of interval 2 to interval 3.**

Interval 1: y = 21.78 – 1.03 time

Interval 2: y = 21.78 – 1.03 time + 42.15 + 6.96 (time – 6) = 22.17 + 5.93 time

Interval 3: y = 21.78 – 1.03 time + 42.15 + 6.96 (time – 6) – 3.84 – 6.67 (time – 14) = 111.71 – 0.74 time

The sudden change after first intervention was significant (sudden change=42.15, p=0.0001). There was a significant change in the slope of the regression after first intervention (change in slope=5.93 – (-1.13) =6.9, p=0.0063). There was also a significant change in the slope of the regression line after 2^nd^ intervention (change in slope= -0.74-(5.93) = -6.67, p=0.0083).

**Outcome C: Percent Accepted Referral =total_accepted_referral/total_offered_referral*100**

| Primary Analyses | DF | Estimate | SE | t Value | Pr > \|t\| |
| --- | --- | --- | --- | --- | --- |
| Intercept 1 | 1 | 63.6243 | 5.4503 | 11.67 | <.0001 |
| Slope 1 | 1 | -6.4342 | 1.3995 | -4.60 | 0.0004 |
| Diff_Intercept_12^a^ | 1 | 12.9453 | 6.2262 | 2.08 | 0.0565 |
| Diff_Slope_12^c^ | 1 | 5.6117 | 1.6658 | 3.37 | 0.0046 |
| Diff_Intercept_23^b^ | 1 | -7.7033 | 6.6324 | -1.16 | 0.2649 |
| Diff_Slope_23^d^ | 1 | -0.1375 | 1.6658 | -0.08 | 0.9354 |

**^a^Diff_Intercept_12 is the difference between the intercepts of interval 1 to interval 2;**

**^b^Diff_Intercept_23 is the difference between the intercepts of interval 2 to interval 3;**

**^c^Diff_Slope_12 is the difference between the slopes of interval 1 to interval 2;**

**^d^Diff_Slope_23 is the difference between the slopes of interval 2 to interval 3.**

Interval 1: y = 63.6243 – 6.4342 time

Interval 2: y = 63.6243 – 6.4342 time +12.9453 + 5.6117 (time – 6) = 42.8994 – 0.8225 time

Interval 3: y = 63.6243 – 6.4342 time + 12.9453 + 5.6117 (time – 6) – 7.7033 – 0.1375 (time – 14) = 37.1211 – 0.96 time

There is a significant downward trend in the 1^st^ interval (slope = -6.4342, p=0.0004). There is also a significant change in slope after 1^st^ intervention (change in slope = -0.8225 – (-6.4342) = 5.6117, p=0.0046).

**Outcome D: Percent Referrals Generated =**

**total_accepted_referral/total_current_smokers_recent_quitter *100**

| Primary Analyses | DF | Estimate | SE | t Value | Pr > \|t\| |
| --- | --- | --- | --- | --- | --- |
| Intercept 1 | 1 | 9.1978 | 3.7487 | 2.45 | 0.0279 |
| Slope 1 | 1 | -1.0293 | 0.9626 | -1.07 | 0.3030 |
| Diff_Intercept_12^a^ | 1 | 15.2879 | 4.2823 | 3.57 | 0.0031 |
| Diff_Slope_12 ^c^ | 1 | 2.1593 | 1.1457 | 1.88 | 0.0804 |
| Diff_Intercept_23 ^b^ | 1 | -7.4450 | 4.5617 | -1.63 | 0.1249 |
| Diff_Slope_23 ^d^ | 1 | -2.1228 | 1.1457 | -1.85 | 0.0851 |

**^a^Diff_Intercept_12 is the difference between the intercepts of interval 1 to interval 2;**

**^b^Diff_Intercept_23 is the difference between the intercepts of interval 2 to interval 3;**

**^c^Diff_Slope_12 is the difference between the slopes of interval 1 to interval 2;**

**^d^Diff_Slope_23 is the difference between the slopes of interval 2 to interval 3.**

Interval 1: y = 9.20 – 1.03 time

Interval 2: y = 9.20 – 1.03 time + 15.29 + 2.16 (time – 6) = 11.53 + 1.13 time

Interval 3: y = 9.20 – 1.03 time + 15.29 + 2.16 (time – 6) – 7.45 – 2.12 (time – 14) = 33.67 – 0.99 time

The sudden change after first intervention was significant (sudden change=15.29, p=0.0031).
